# Among the Masses: Multiple Left Atrial Undifferentiated Pleomorphic Sarcomas

**DOI:** 10.31486/toj.23.0143

**Published:** 2024

**Authors:** Kevin Yager, Jerry Fan, Corry B. Sanford, Niloufar Pourfarrokh, Vinh Nguyen

**Affiliations:** ^1^ Department of Internal Medicine, Baylor Scott & White Medical Center, Temple, TX; ^2^ Division of Cardiology, Baylor Scott & White Medical Center, Temple, TX; ^3^ Department of Pathology, Baylor Scott & White Medical Center, Temple, TX

**Keywords:** *Heart neoplasms*, *immunotherapy*, *left atrial mass*, *pleomorphic sarcoma*

## Abstract

**Background:** Undifferentiated pleomorphic sarcoma, an exceedingly rare and aggressive primary cardiac tumor arising from mesenchymal stem cells, is associated with poor prognosis and high mortality despite adequate treatment.

**Case Report:** A 52-year-old female presented with a 2-month history of angina and dyspnea on exertion. Her clinical history included severe acute respiratory syndrome coronavirus 2 myocarditis and iron deficiency anemia. Elevated troponin 1 and D-dimer prompted further investigation, and diagnostic imaging revealed multiple hypodensities in the left atrium and a right-sided pleural effusion that were causing severe mitral stenosis and pulmonary hypertension. Full body positron emission tomography scan suggested metastatic disease, prompting surgical resection of the atrial masses. Pathology confirmed high-grade undifferentiated pleomorphic sarcoma. Treatment with chemotherapy resulted in clinical stability without radiographic evidence of recurrence at 9 months. At follow-up >2 years after the initial diagnosis, echocardiogram demonstrated normal left ventricular systolic function with ejection fraction of 55% to 60%, no mitral gradient, and resolution of pulmonary hypertension.

**Conclusion:** Left atrial masses are a diagnostic challenge because of the location and the technical difficulty of biopsy. Undifferentiated pleomorphic sarcoma has a 5-year survival rate of approximately 60%, so the condition necessitates prompt diagnosis and treatment.

## INTRODUCTION

Primary cardiac tumors are exceedingly rare, representing 0.0017% to 0.33% of tumors; most tumors found in the heart are metastatic tumors.^1^ Undifferentiated pleomorphic sarcoma is a rare type of primary cardiac tumor that usually arises from the left atrium and often involves the mitral valve, causing heart failure.^2^ Survival is often poor (<1 year) because of advanced disease at the time of diagnosis.^3^ Although there is no standard of treatment for undifferentiated pleomorphic sarcoma, surgical resection can be curative if the tumor is isolated and complete resection is achieved, but in most cases, metastatic disease will be treated with a combination of surgery, chemotherapy, and radiation.^2,4^

We present a case of primary left atrial undifferentiated pleomorphic sarcoma with small bowel metastasis with >2-year survival after therapy with pembrolizumab.

## CASE REPORT

A 52-year-old female with a history of obesity, Crohn disease with hemicolectomy, obstructive sleep apnea, transfusion-dependent anemia (because of heavy menstrual cycles), and severe acute respiratory syndrome coronavirus 2 myocarditis presented with a 2-month history of angina and dyspnea on exertion. Laboratory profile revealed elevated troponin 1 (0.30 ng/mL, repeat 0.24 ng/mL; reference range, 0.00-0.09 ng/mL) and D-dimer (1.98 μg/mL; reference range, 0.00-0.50 μg/mL).

**Figure 1.**
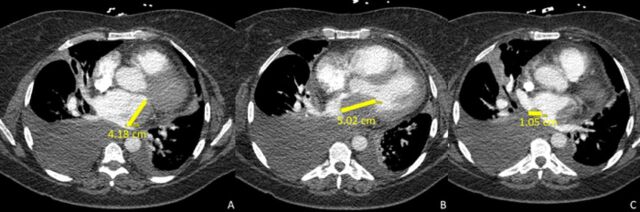
(A, B, C) Computed tomography shows 3 distinct left atrial masses adherent to the left atrium (measuring 4.18 cm, 5.02 cm, and 1.05 cm) with right-sided pleural effusion.

Chest computed tomography (CT) ruled out pulmonary embolism but was notable for multiple hypodensities in the left atrium measuring 4.18 cm, 5.02 cm, and 1.05 cm and a moderately sized right-sided pleural effusion (Figure 1). Transthoracic echocardiogram visualized 2 masses in the left atrium measuring 4.3 cm × 2.8 cm and 3.5 cm × 2.0 cm that were causing severe functional mitral stenosis and pulmonary hypertension (peak mitral inflow velocity 321 cm/s, mean gradient 20 mm Hg at 79 beats per minute, pulmonary artery systolic pressure 63 mm Hg).

**Figure 2.**
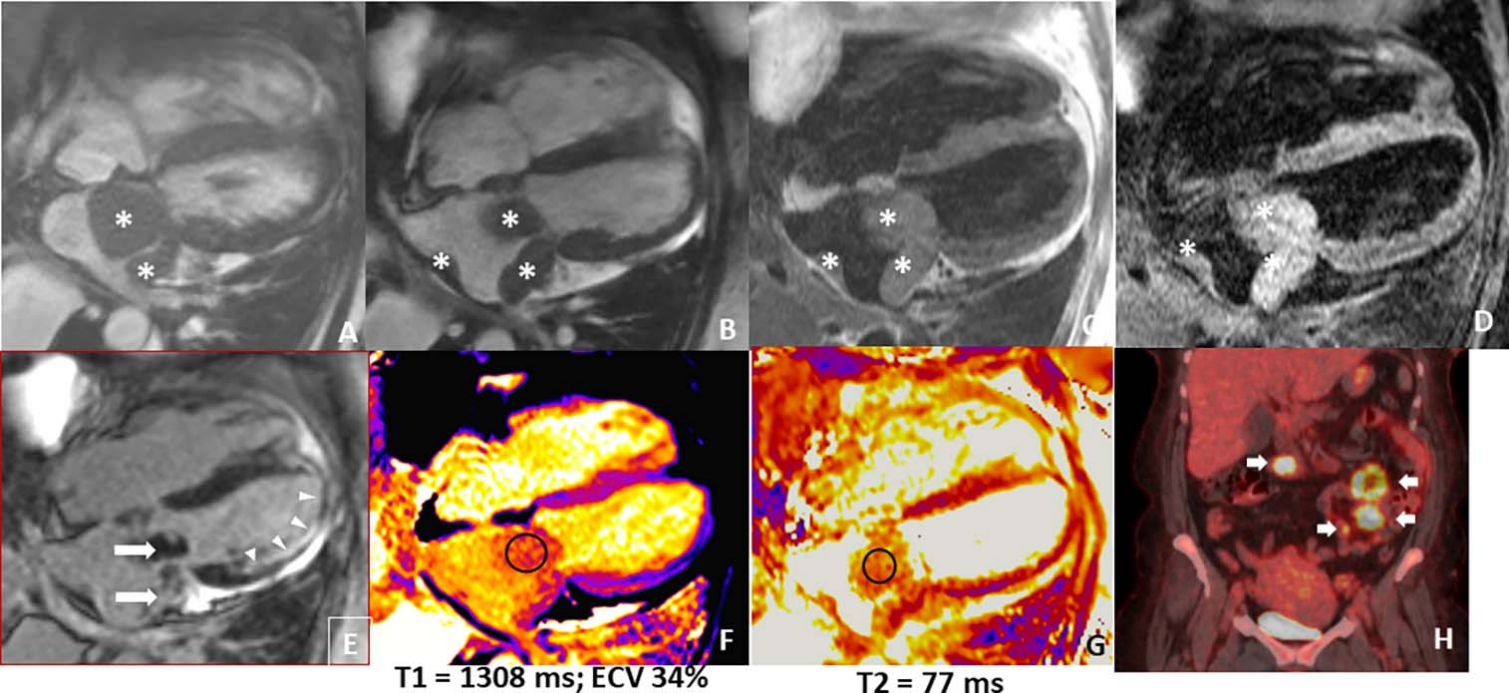
**(A, B) Steady-state free precession magnetic resonance imaging sequence shows multiple left atrial masses, with the largest mass in relation to the fossa ovalis and the mitral valve. (C) Isointense signal to the myocardium on T1-weighted imaging. (D) Hyperintense signal to the myocardium on T2-weighted imaging. (A, B, C, D) Asterisks show the locations of the left atrial masses. (E) Heterogenous late gadolinium enhancement (arrows) and incidental infarct pattern at the lateral and apical segments (arrowheads). (F, G) Parametric mapping with increased T1 signal suggests inflammation, and increased T2 signal is consistent with edema. The circles represent the relaxation time measurement in the tumor on T1- and T2-weighted imaging which demonstrates the high fluid and interstitial content within the tumor. (H) Positron emission tomography scan shows multiple hypermetabolic foci (arrows).** ECV, extracellular volume; ms, milliseconds.

Cardiac magnetic resonance imaging (MRI) showed multiple masses in the left atrium. MRI favored a diagnosis of left atrial myxoma because of tissue characterization of the mass as isointense to the myocardium on T1-weighted imaging and hyperintense to the myocardium on T2-weighted imaging, suggesting high fluid content and high interstitial content that are most consistent with left atrial myxoma vs other cardiac tumors (Figure 2). Interventricular septal flattening in systole was consistent with severe pulmonary hypertension.

Full body positron emission tomography (PET) scan showed multiple small bowel lesions and mesenteric implants suggestive of metastatic disease. The standardized uptake values of the small bowel lesions when compared to the cardiac lesions were suggestive of a primary cardiac tumor with metastasis to the small bowel.

**Figure 3.**
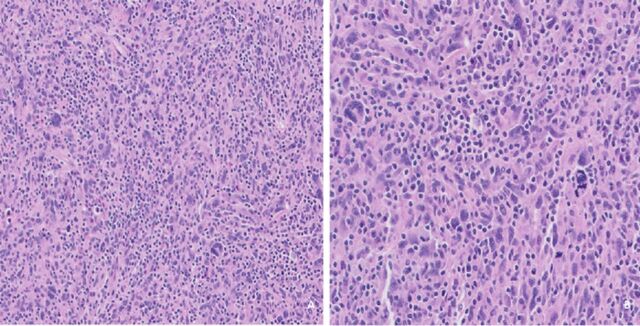
(A) Hematoxylin and eosin [H&E] stain, magnification ×10, and (B) H&E stain, magnification ×20 show spindle to ovoid and markedly pleomorphic tumor cells admixed with marked inflammatory cells, including numerous plasma cells and a few scattered eosinophils. Numerous mitoses, including atypical mitoses, are present.

A diagnosis of primary cardiac malignancy was favored after review by 3 independent cancer centers (Baylor Scott & White Medical Center [our home institution], Mayo Clinic, and MD Anderson) based on the available data. The patient underwent surgical excision of the atrial masses. Pathology revealed high-grade undifferentiated pleomorphic sarcoma (Figure 3).

The patient underwent suppressive chemotherapy with ifosfamide (10 mg/m^2^) and doxorubicin (75 mg/m^2^). After 4 cycles, she developed significant emotional instability manifesting as periods of delirium, severe agitation, hallucinations, and confusion, known complications associated with ifosfamide. The patient was transitioned to a non-ifosfamide regimen that included doxorubicin (75 mg/m^2^), dexrazoxane (750 mg/m^2^), and dacarbazine (750 mg/m^2^) for 3 cycles.

Follow-up echocardiogram demonstrated the development of cardiomyopathy (ejection fraction of 65% to 70% decreased to 41% to 45% during a 6-month period), and the patient was transitioned to pembrolizumab for immunotherapy.

**Figure 4.**
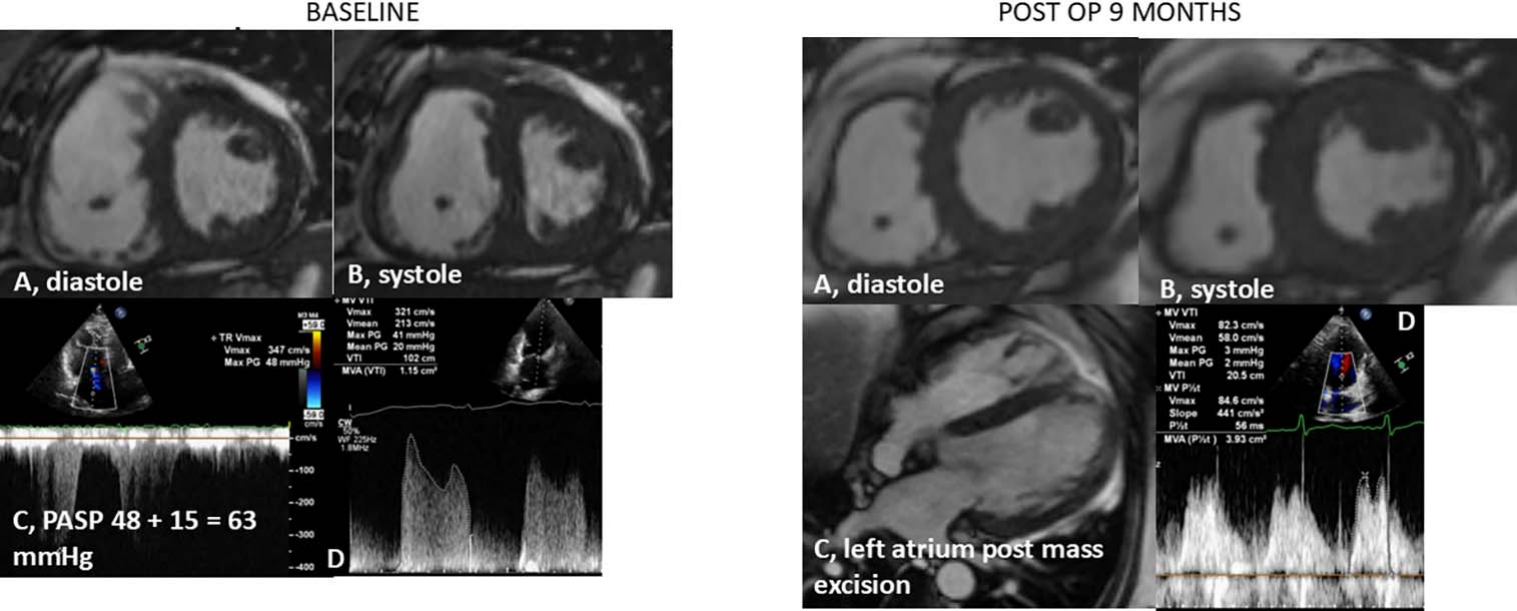
Left panel, baseline imaging: (A) Preoperative cardiac magnetic resonance imaging (MRI) in diastole. (B) Preoperative cardiac MRI in systole shows a D-shaped septum consistent with pulmonary hypertension from functional mitral stenosis. (C) Echocardiography shows pulmonary artery systolic pressure (PASP) of 48 mm Hg. (D) Echocardiography shows PASP elevated at 63 mm Hg, peak mitral inflow velocity of 321 cm/s, and mean gradient of 20 mm Hg. Right panel, postoperative imaging: (A) Postoperative cardiac MRI in diastole. (B) Postoperative cardiac MRI in systole shows resolution of the D-shaped septum. (C) Cardiac MRI of the left atrium after excision of the atrial masses. (D) Echocardiography shows postoperative normalization of the peak transmitral velocity and mean gradient.

Nine months after the initial resection, cardiac MRI showed no residual cardiac mass. Echocardiogram showed an interval decrease in transmitral mean gradient from baseline 20 mm Hg to 2 mm Hg, and interventricular septal flattening was no longer present (Figure 4). The patient's severe pulmonary hypertension was thought to have been caused by functional mitral stenosis from the mass.

At follow-up >2 years after the initial diagnosis, echocardiogram demonstrated normal left ventricular systolic function with ejection fraction of 55% to 60%, no mitral gradient, and resolution of pulmonary hypertension. Surveillance CT of the chest, abdomen, and pelvis demonstrated 1 mesenteric mass measuring 1.5 × 1.3 cm (decreased from 2.3 × 2.1 cm, 6 months prior) after the patient had completed 1 year of pembrolizumab.

## DISCUSSION

Undifferentiated pleomorphic sarcoma is an aggressive soft tissue tumor that arises from mesenchymal stem cells.^5,6^ The mechanism of tumorigenesis involves a complex interplay of multiple altered genomics that remains poorly understood despite advancements in identification and gene sequencing.^6^ The incidence of undifferentiated pleomorphic sarcoma favors males over females, White males over Black males, and increasing age, especially beyond the sixth decade of life.^7^

Left atrial undifferentiated pleomorphic sarcoma is a rare subtype occurring in 1% of cases.^8^ Primary cardiac tumors often remain clinically asymptomatic until sequelae of mass effect or nonspecific symptoms (fatigue, fever, weight loss, night sweats) prompt evaluation; consequently, these tumors are a challenge to diagnose and treat.^2,6,9,10^ These tumors are often mistaken for benign cardiac tumors such as atrial myxomas during initial evaluation because of their similar clinical and imaging presentations; however, undifferentiated pleomorphic sarcoma often presents with multiple masses rather than a single mass.^11,12^ Left atrial undifferentiated pleomorphic sarcoma can invade the left atrial and ventricular walls, leading to mitral valve dysfunction and eventual left-sided heart failure with dyspnea, chest pain, or embolic phenomena.^2,10,11^

The diagnosis of left atrial undifferentiated pleomorphic sarcomas is also challenging because of their heterogeneous nature and the lack of specific diagnostic tests. Typically, a combination of imaging studies, including echocardiography, CT scan, MRI, and PET scan are used to detect the presence of a left atrial undifferentiated pleomorphic sarcoma.^6,13^ Although echocardiography is often used as a first-line imaging study for cardiac tumors, it has distinct disadvantages. Limited field of view, the inability to characterize tissue, and factors such as body habitus and patient cooperation limit the utility of echocardiography in the diagnosis and evaluation of cardiac tumors.^13^ The National Comprehensive Cancer Network recommends CT or MRI as the imaging modality of choice to evaluate for tumor-node-metastasis staging, along with image-guided core biopsy.^14^ Cardiac MRI offers distinct advantages over echocardiography, including excellent spatial resolution, wide field of view, the relationship of the tumor with structures outside the heart, and tissue characterization which allows for identification of cardiac tumors that do not need further evaluation or resection.^13^ However, imaging studies may not be specific enough to distinguish between left atrial undifferentiated pleomorphic sarcoma and other cardiac tumors such as myxomas.^2^ In the initial cardiac MRI evaluation of our patient, we suspected the cardiac tumor to be a left atrial myxoma because of the tissue characterization on T1- and T2-weighted imaging. Specifically, the mass was isointense to the myocardium on T1-weighted imaging and hyperintense to the myocardium on T2-weighted imaging, suggestive of high fluid content and high interstitial content that are most consistent with left atrial myxoma vs other cardiac tumors. The increased T1 relaxation time was likely attributable to a combination of inflammation and fibrosis. Classic findings on multimodality imaging that favor malignancy include uptake of contrast, infiltration and invasion of normal anatomic boundaries, and the presence of necrosis.^12^

Histopathology is paramount in the diagnosis of undifferentiated pleomorphic sarcoma; a combination of microscopy and immunohistochemical markers should be used for diagnosis.^6^ Undifferentiated pleomorphic sarcoma under microscopy demonstrates “atypical, pleomorphic spindle cells with abundant mitotic figures.”^6^ Tumor proteins such as Dickkopf-related protein 1, tumor protein p53, cyclin-dependent kinase inhibitor 2A, retinoblastoma-associated protein, and transcriptional regulator ATRX are all possible causes of tumorigenesis.^6^

The treatment of left atrial undifferentiated pleomorphic sarcoma is complex and requires a multidisciplinary team. Surgery is the primary treatment option with the goal of achieving complete resection.^4,6,10,12,15^ Chemotherapy and radiation therapy are used as adjuvant therapies for unresectable tumors; however, in cases of extensive metastasis or advanced disease, these therapies are often palliative and not curative.^4,6,9,15^ There is no standard treatment protocol for left atrial undifferentiated pleomorphic sarcoma, and the optimal treatment approach is individualized based on the patient's clinical presentation, tumor size, and location.^6,9,10^

Interestingly, in a study of undifferentiated pleomorphic sarcomas, histology-guided chemotherapy was not superior to standard chemotherapy regimens (anthracycline + ifosfamide) in advanced-stage tumors.^16^ New immunotherapies, including pembrolizumab, nivolumab, and ipilimumab, have been used as alternatives to traditional chemotherapy for treatment of undifferentiated pleomorphic sarcoma but are currently considered investigational.^6,15^

Malignant tumors of the heart in general have a poor prognosis with a 1-year survival rate of approximately 10%.^13^ The prognosis of undifferentiated pleomorphic sarcoma is poor overall, with Vodanovich et al reporting a 5-year survival rate of approximately 60%.^17^ Primary cardiac undifferentiated pleomorphic sarcoma has a considerably worse outcome with a median survival of <1 year.^4,10-12^ Postoperative complications are common after resection because of the long bypass time, and the rate of 30-day mortality after resection is 14% to 22.7%, usually as a result of heart failure.^2^ Recurrence rates for undifferentiated pleomorphic sarcoma are generally higher with advanced stages, large primary tumors, and deep invasion of surrounding tissue.^17,18^

This case report represents 1 of a handful of reported cases of longer-term survival using new immunotherapies for the treatment of left atrial undifferentiated pleomorphic sarcoma.^14^ Most cases of left atrial undifferentiated pleomorphic sarcoma have a median survival <1 year with standard chemotherapy regimens and surgical resection.^14,17,18^ Our patient was previously on standard therapy with anthracycline and ifosfamide but developed cardiomyopathy, necessitating a switch to immunotherapy for approximately 1 year with continued remission of disease.

## CONCLUSION

Left atrial undifferentiated pleomorphic sarcoma is a rare type of primary cardiac tumor with significant morbidity and mortality. Diagnosis is a challenge because of the nonspecific clinical presentation, tissue characterization that can mimic other primary cardiac tumors, and lack of effective treatment options. Because of the toxicity and side effects of first- and second-line treatment options, our patient was transitioned to investigational therapy (immunotherapy with pembrolizumab), resulting in >2-year survival. Further research is needed to evaluate long-term survival rates and quality of life outcomes in patients treated with immunotherapy for left atrial undifferentiated pleomorphic sarcoma to better understand the disease course and optimization of patient care for this rare disease state.

## Notes

*This article meets the Accreditation Council for Graduate Medical Education and the American Board of Medical Specialties Maintenance of Certification competencies for Patient Care and Medical Knowledge.*
